# Identifying hidden infection risks in urinary catheterisation: a nurse-led quality improvement initiative based on clinical observation

**DOI:** 10.1016/j.infpip.2026.100577

**Published:** 2026-08-07

**Authors:** Erzsébet Horváthné Konya, Zoltán Balogh

**Affiliations:** a Semmelweis University, Faculty of Health Sciences, Department of Nursing, Budapest, Hungary; b Semmelweis University, Doctoral College Semmelweis University, Budapest, Hungary

## Introduction

Catheter-associated urinary tract infection (CAUTI) remains one of the most common healthcare-associated infections and continues to be associated with increased morbidity, longer hospital stay, additional antimicrobial treatment, and avoidable healthcare costs [1,2]. Because a substantial proportion of hospital-acquired urinary tract infections occur in catheterised patients, urinary catheterisation remains a central concern in infection prevention practice. International guidance has consistently emphasised appropriate catheter indication, aseptic insertion, maintenance of a closed drainage system, secure fixation, unobstructed urine flow, and timely catheter removal [3]. These principles are well established and remain essential. However, the practical delivery of urinary catheterisation at the bedside may involve additional procedural vulnerabilities that are less visible in surveillance data and less fully articulated in guideline-level recommendations.

In routine nursing practice, catheterisation is not performed in idealised conditions alone. It is carried out in real care environments shaped by time pressure, patient dependency, anatomical difficulty, equipment availability, staffing constraints, and variable procedural support. Within these conditions, nurses may repeatedly encounter small but clinically relevant problems that are not always captured in incident reports or formal infection surveillance. Such problems may include incomplete lubrication, difficulty maintaining sterility during single-operator insertion, anatomical challenges affecting visualisation, catheter movement after insertion, and variability in urine sampling practice.

This paper does not present a formal epidemiological or interventional study. Instead, it provides a practice-based account of recurring observations derived from routine nursing care and educational supervision in urinary catheterisation, reflecting clinical experience from Hungary. By presenting these observations transparently, the paper aims to illustrate how bedside nursing insights may inform quality improvement in CAUTI prevention.

## Available knowledge

The current literature identifies several well-established contributors to CAUTI risk. These include inappropriate catheter use, prolonged catheterisation, breaches in aseptic technique, breaks in the closed drainage system, inadequate catheter maintenance, and failures in timely removal [4]. Prevention strategies therefore focus on minimising catheter use, ensuring aseptic insertion, maintaining system integrity, and standardising catheter care. The literature also recognises that urethral trauma and repeated manipulation may have infection-relevant consequences. Mechanical irritation of the urethral mucosa can compromise local barriers, and handling of the catheter or drainage system may facilitate microbial entry if aseptic conditions are not maintained. Similarly, urine specimen collection from a catheterised patient is recognised as an infection-sensitive procedure, particularly when manipulation of the drainage system is required.

Large surveillance systems provide important epidemiological insight into the burden and distribution of healthcare-associated urinary tract infections, but they usually do not capture the detailed procedural realities of individual catheterisation encounters [5]. As a result, fine-grained bedside issues may remain underdescribed, particularly when they occur intermittently, are shaped by local context, or are recognised primarily through repeated nursing observation rather than formal event reporting.

Published guidance is strong on the principles of infection prevention, but there is less detailed discussion of how these principles are challenged in routine bedside execution, especially in situations involving single-operator practice, incomplete sterile equipment, visually difficult anatomy, or uncertainty regarding urine sampling methods. This creates space for reflective practice reports that do not claim causal proof but that can still identify recurring vulnerabilities relevant to quality improvement.

## Rationale

Frontline nurses occupy a distinctive position in relation to urinary catheterisation. They perform the procedure, supervise it, observe its practical difficulties, and often recognise procedural vulnerabilities before those vulnerabilities are formalised in guidelines, audits, or surveillance frameworks. In this sense, nursing observation may function as an early detector of quality and safety problems. In our clinical and educational experience, urinary catheterisation repeatedly raised concerns that were not limited to classical descriptions of aseptic breach. Recurring observations included difficulty achieving uniform lubrication, maintaining sterility during single-operator catheterisation, identifying the urethral meatus in some female patients, preventing unintended inward movement of the catheter after insertion, and performing urine sampling consistently when recommended equipment was not uniformly available. These observations do not establish frequency, effect size, or causality. They are not presented as outcome data and should not be interpreted as such. Their value lies instead in identifying recurrent practice patterns that may warrant closer scrutiny in nursing education, protocol development, infection prevention strategy, and nurse-led innovation. A transparent account of these recurring observations may therefore contribute to more context-sensitive quality improvement in urinary catheterisation practice [6].

## Aim

This paper describes recurring bedside practice observations related to urinary catheterisation that may contribute to procedural difficulty and infection risk. It further considers how these observations may inform nurse-led quality improvement and practice-driven innovation in the prevention of CAUTI.

## Methods

This manuscript is presented as a reflective nurse-led quality improvement report based on structured clinical observation. It is not a formal prospective, retrospective, interventional, mixed-methods, or hypothesis-testing research study. It does not report a predefined sample, formal recruitment, statistical testing, or a designed qualitative dataset.

## Context and setting

The observations described in this report arose from routine urinary catheterisation practice and educational supervision in Hungary across a range of clinical care settings over approximately the past 25 years. These settings included chronic internal medicine, geriatric care, hospice care, and, for 2.5 years during the coronavirus disease 2019 pandemic, active inpatient care in high-risk isolation (“red zone”) conditions. The focus of the report is the bedside performance of urinary catheterisation and related infection-control practices.

## Source of observations

The observations were derived from both direct bedside care and educational supervision. They reflect recurring patterns recognised across routine catheterisation encounters rather than data collected through interviews, surveys, focus groups, or a formal observational instrument. The observations were synthesised retrospectively from repeated clinical experience accumulated by a Hungarian registered nurse with nearly 25 years of professional practice. Over the past 15 years, this clinical experience was further complemented by continuous academic development, including BSc and MSc qualifications, and is currently informed by ongoing doctoral studies as a PhD student. The combination of long-term frontline clinical work, sustained professional education, and progressive expansion of clinical and academic perspective contributed to the reflective analysis presented in this report.

## Nature of analysis and reflection

The manuscript organises recurring practice observations into clinically meaningful themes relevant to infection prevention and nursing quality improvement. No formal checklist, validated scoring tool, coding software, interview framework, or statistical analysis was used. The analysis was descriptive and interpretive. Its aim was to identify repeated bedside concerns and to distinguish, as clearly as possible, between what was directly observed in practice and what may reasonably be inferred in relation to infection risk [7].

## Results

Across routine urinary catheterisation encounters, recurring practice observations suggested that infection-relevant vulnerabilities extended beyond broadly stated aseptic principles alone. Structured bedside reflections identified six interrelated themes: lubrication-related practice issues, maintaining sterility during single-operator catheterisation, anatomical and sex-related challenges, catheter migration, urine sampling variability, and environmental or organisational contributors.

## Theme 1. Lubrication-related practice issues

Recurring practice observations suggested that lubrication during catheter insertion was often uneven, insufficient, or functionally misdirected. In everyday care, commonly used techniques included coating the catheter tip, applying lubricant along the external surface of the catheter, or using gel-covered gauze. Although these methods created the appearance of preparation, they did not always ensure effective lubrication of the urethral lumen itself, particularly in female catheterisation, where the lubricant was often applied primarily to the device rather than delivered into the urethra. As a result, the internal mucosal surfaces through which the catheter had to pass frequently remained inadequately lubricated.

This issue is clinically important because the urethra is not a smooth, regular cylindrical channel into which a catheter can be advanced without resistance. Rather, it consists of closely apposed mucosal tissues, with possible anatomical irregularities, folds, tissue fragility, or scar-related unevenness resulting from previous irritation, trauma, inflammation, or repeated instrumentation. Under such conditions, lubricant applied only to the catheter surface may be rapidly displaced or wiped away during insertion, particularly as the catheter passes through narrower or more resistant segments, including the external urethral sphincter. The direct observation was therefore not simply variability in lubrication practice but a recurring mismatch between where lubrication was applied and where it was actually needed.

The inferred concern was that inadequate lubrication of the urethral passage may increase friction against the mucosa, contribute to microtrauma, and reduce patient comfort. From an infection-prevention perspective, such repeated or avoidable mucosal injury may plausibly weaken local epithelial protection and facilitate microbial entry. These observations were particularly relevant in technically difficult insertions, where resistance, repositioning, or repeated manipulation occurred.

A further practical concern related to the use of lubricants containing local anaesthetic agents. When such products were applied mainly to the catheter surface rather than into the urethra itself, their intended effect on patient comfort was likely to be substantially reduced. In functional terms, the anaesthetic component was acting more on the device than on the patient. This observation raised concern that current bedside lubrication practices may sometimes fail in both respects: they may provide inadequate mucosal lubrication and limited analgesic benefit at the very site where these effects are most needed.

Taken together, these reflections suggest that lubrication should not be regarded as a minor preparatory detail but as a clinically important component of atraumatic, patient-centred, and infection-conscious catheterisation practice [8].

## Theme 2. Maintaining sterility during single-operator catheterisation

Structured bedside reflections identified repeated difficulty in maintaining sterility when catheterisation was performed by a single operator, especially in settings where a complete single-use sterile catheterisation set was unavailable. In these circumstances, nurses often had to prepare equipment, maintain exposure of the perineal area, manage lubricant, preserve sterility, and insert the catheter within a narrow sequence of actions. The direct observation was that single-operator catheterisation required simultaneous management of multiple sterile and non-sterile tasks. This was especially demanding in patients who were immobile, in pain, restless, cognitively impaired, or difficult to position. The inferred concern was that such a workflow increased the likelihood of accidental contamination even when the nurse understood and intended to follow aseptic principles correctly.

These observations suggest that deviations from ideal sterile technique may not always arise from inadequate knowledge or poor professional commitment. In some cases, they reflect the practical limits of a complex procedure performed under routine bedside conditions without optimal equipment or assistance. This theme therefore points toward workflow design and resource availability as relevant components of infection prevention.

## Theme 3. Anatomical and sex-related challenges

Recurring observations indicated that anatomical conditions influenced both procedural difficulty and contamination risk. In female patients, visualisation of the urethral meatus was often more difficult in the presence of obesity, immobility, incontinence, oedema, poor lighting, limited positioning options, or reduced patient cooperation. The short urethra and the close anatomical proximity of the urethral meatus to the anal sphincter, vaginal introitus, and surrounding perineal structures made preservation of a clean working field more difficult during insertion. Direct observation indicated increased technical difficulty in locating and accessing the urethral opening in some female patients. This sometimes resulted in prolonged attempts, repeated handling, the need for repositioning, or inadvertent insertion of the catheter into the vagina during the first attempt. A further recurring concern was that, in some cases, a catheter that had initially entered the vaginal canal was not replaced with a new sterile device but was subsequently redirected and inserted toward the urethra. From an infection-prevention perspective, this represented a particularly important vulnerability, as a device that had already contacted non-target mucosal surfaces was being reintroduced into the urinary tract. The inferred implication was that extended manipulation, repeated attempts, and reuse of a misdirected catheter may increase both contamination risk and patient discomfort. These observations do not imply that female sex alone determines infection outcome. Rather, they suggest that anatomical and physiological conditions relevant to catheterisation may require greater procedural sensitivity than some standard protocols explicitly acknowledge. In postmenopausal patients, additional mucosal fragility and altered local microbiological conditions may further contribute to procedural vulnerability, although the present report does not attempt to measure these factors directly. The practical implication of this theme is that catheterisation should be approached as an anatomy-sensitive procedure, particularly in patients where visualisation is poor, tissue fragility is expected, or repeated attempts increase the risk of contamination.

## Theme 4. Catheter migration

One recurring bedside concern was unintended inward catheter migration after insertion. By this term, we refer to situations in which the external portion of an indwelling catheter, already exposed to skin, clothing, bed linen, incontinence material, or body fluids, appeared to shift proximally and re-enter further toward the urethral tract. The direct observation was a change in the visible length or position of the catheter during routine care, including patient repositioning, hygiene care, absorbent product changes, or handling of the device by the patient. In some cases, nurses first noticed this through altered visible catheter length; in others, it was recognised during catheter care or because of unexpected changes in catheter positioning or urine flow [9].

From a nursing and infection-prevention perspective, this raised a particularly important concern: a catheter segment that had already been exposed to potentially contaminated external surfaces could subsequently move back toward the urethral opening. The inferred implication was that inward displacement of such an external segment may bring micro-organisms closer to, or into, the urethral tract. This concern appeared especially relevant when the urethral mucosa may already have been irritated or rendered more vulnerable by friction, inadequate lubrication, repeated manipulation, or technically difficult insertion. In such cases, catheter migration may represent not merely a matter of device position or patient comfort but a clinically meaningful mechanism by which contamination risk could be increased during routine daily care.

These observations also suggested that post-insertion catheter management deserves greater attention than is often reflected in simplified procedural descriptions. While insertion technique remains central, the infection-prevention implications of what happens to the catheter after placement, including fixation quality, routine handling, patient movement, hygiene care, and repositioning, may be equally important. From this perspective, catheter migration should be considered not only a mechanical issue but also a potential infection-control concern requiring closer attention in bedside practice, staff training, and device-related quality improvement.

## Theme 5. Urine sampling variability and infection-control concerns

Bedside experience also showed that urine sampling practice in catheterised patients was not always uniform. Although specimen collection is expected to follow aseptic principles, the equipment available and the method used were not always consistent across care settings. This created uncertainty around a task that should ideally be standardised and low risk.

This issue became particularly relevant in light of the Hungarian national professional guideline published in July 2025 by the Ministry of Interior on nursing tasks related to high-risk invasive procedures. In the section addressing urine sampling from an indwelling urinary catheter, the guideline states that the specimen should be obtained using aseptic technique and that, in the case of a permanent catheter, the catheter wall should be punctured with a sterile needle attached to a sterile syringe above the junction with the drainage tubing, or at the designated area intended for this purpose, after prior disinfection [10]. The same guidance also clearly states that the sample must not be taken from the drainage bag. However, this creates a practical and regulatory tension. While the Hungarian national guideline describes catheter wall puncture as an option for urine sampling from an indwelling catheter, device-specific instructions for use may prohibit puncturing the catheter wall. This discrepancy has previously been described as a safety paradox in urinary catheterisation, where national procedural guidance may conflict with manufacturer instructions for use [11]. Such inconsistency may leave nurses uncertain about which instruction should take priority in bedside practice.

In routine care, this was reflected in recurring uncertainty about exactly where and how the sample should be taken. In some cases, bladder urine was not obtained because balloon inflation fluid was aspirated instead, creating a risk of laboratory error and clinical misinterpretation. The timing of specimen collection was also not always guided by a clearly harmonised local protocol. Taken together, these observations suggest that urine sampling in catheterised patients should not be treated as a minor technical step but as an infection-sensitive procedure requiring clearer alignment between national guidance, local protocols, and the equipment actually available at the bedside.

## Theme 6. Environmental and organisational contributors

Across routine catheterisation encounters, the above themes appeared to be shaped by broader organisational conditions. Nurses often worked in environments characterised by time pressure, variable staffing, limited bedside space, inconsistent lighting, and unequal access to complete sterile equipment. Training and supervision also appeared to vary between settings and practitioners. A particularly important contextual factor was workforce shortage, which directly influenced the conditions of everyday nursing practice. This is also consistent with the Hungary: Country Health Profile 2025, part of the joint OECD and European Commission country profile series, which highlights persistent health workforce challenges in Hungary [12]. The direct observation was that procedural difficulty rarely occurred in isolation. Rather, it was embedded in care environments that made ideal practice harder to achieve consistently. Under these conditions, even experienced nurses could face increased risk of minor deviations, repeated manipulation, or compromised workflow.

The inferred implication was that CAUTI prevention should not be framed only as a matter of individual technical competence. It is also influenced by system-level factors, including the availability of appropriate equipment, the design of workflows, the quality of procedural training, and the extent to which repeated bedside concerns are recognised and addressed at institutional level. Where workforce shortages are persistent, maintaining optimal infection-prevention practice becomes not only a matter of knowledge and skill but also of capacity.

## Discussion

This reflective nurse-led quality improvement report describes recurring bedside observations suggesting that infection-relevant vulnerabilities in urinary catheterisation extend beyond simplified descriptions of aseptic technique alone. The observations clustered around six themes: incomplete or unstable lubrication, difficulty maintaining sterility during single-operator procedures, anatomy-related catheterisation difficulty, unintended inward catheter migration, variability in urine sampling technique, and organisational conditions shaping procedural performance. These themes are not presented as quantified causal evidence. Rather, they are presented as structured recurring practice observations that may help identify overlooked areas for quality improvement. Their significance lies in showing that urinary catheterisation, although often treated as a routine procedure, may contain multiple small but clinically meaningful vulnerabilities that are easily normalised in everyday practice.

## Interpretation in relation to literature

The present observations are broadly consistent with the existing infection-prevention literature, which emphasises aseptic insertion, minimisation of urethral trauma, maintenance of a closed system, and careful handling of the catheter and drainage apparatus [13]. However, the current report extends that discussion by drawing attention to how these principles are operationally challenged under real bedside conditions. The lubrication-related observations support the broader understanding that atraumatic insertion matters not only for comfort but also for preservation of mucosal integrity. The findings concerning urine sampling likewise align with infection-control concerns regarding breaches in system integrity and inconsistency in specimen collection practice. Although urine sampling is often treated as a routine technical step, the bedside reflections in this report suggest that it may function as a high-risk infection-control event when equipment and guidance are not fully aligned [14]. The catheter migration theme is less often foregrounded in standard descriptions of CAUTI prevention. Yet from a nursing perspective, it may represent an under-recognised mechanism by which a contaminated external device surface is brought back toward the urethral tract. This report does not prove that such migration causes infection, but it does suggest that the phenomenon is clinically plausible and worthy of more explicit consideration in catheter care, fixation practice, and future quality-improvement work. The anatomical observations should also be interpreted carefully. The report does not argue for a deterministic or reductionist account of sex-based infection risk. Instead, it suggests that certain anatomical and physiological conditions may increase procedural difficulty and therefore deserve more explicit attention in training, practical guidance, and bedside planning.

## Implications for nursing practice and quality improvement

Several practical implications arise from these observations. First, urinary catheterisation should be understood not merely as a technical skill but as an infection-sensitive process requiring anticipatory nursing judgement. Safe performance depends not only on knowing the correct sequence of steps but also on recognising where contamination, trauma, or device-related problems are most likely to occur in a given patient and setting. Second, education and competency development should address real-world bedside difficulty, not only idealised procedural standards. Training may need to place greater emphasis on challenging anatomy, lubrication adequacy, sterile workflow under single-operator conditions, secure fixation, post-insertion catheter handling, and safe urine sampling decision-making. Third, the findings support stronger standardisation of materials and workflows. Access to complete sterile catheterisation sets, clearly defined specimen collection protocols, and alignment between institutional guidance and device instructions for use may reduce avoidable variability in practice. Fourth, this report highlights the importance of structured feedback loops through which repeated bedside concerns can inform institutional quality improvement. Nurses are often the first to detect recurring procedural vulnerabilities. When these observations remain informal, system-level learning is limited. When they are documented, thematically analysed, and discussed, they can contribute to more responsive infection-prevention strategies and practice-driven innovation. More broadly, the paper supports the legitimacy of nurse-led clinical observation as a starting point for quality improvement, provided its methodological boundaries are stated clearly and its claims remain proportionate to the nature of the evidence.

## Limitations

This manuscript should be interpreted as a reflective quality improvement report and not as a formal study designed to determine prevalence, association, or intervention effect. No predefined sample, formal observational instrument, qualitative interview dataset, or statistical analysis was used. The report therefore cannot establish how often these issues occurred, cannot link them directly to measured CAUTI outcomes, and cannot determine causality.

At the same time, the strength of the paper lies in its close proximity to routine practice. It captures recurring procedural concerns recognised through bedside nursing care and educational supervision, many of which are biologically and procedurally plausible in light of current infection-prevention knowledge. Its contribution is therefore not formal proof, but disciplined visibility: making real nursing observations explicit, structured, and usable for quality-improvement thinking.

In conclusion, urinary catheterisation is often treated in everyday practice as a routine and familiar procedure, yet bedside experience shows that it deserves far greater attention. This professional summary highlights that catheterisation involves several recurring issues that may appear minor at first glance but are in fact important from an infection-prevention perspective and can easily become normalised in daily practice. These include the adequacy of lubrication, maintenance of sterility, anatomy-sensitive insertion, subsequent catheter displacement, variability in urine sampling practice, and the organisational conditions in which catheterisation is actually performed. This report does not aim to provide causal proof, nor does it present quantified outcome data. Its value lies in making visible those recurring professional concerns that reappear at the bedside and that deserve greater attention in practice, education, infection prevention, and quality improvement. The nursing perspective is especially important in this field because it is the nurse who sees, performs, and evaluates the real conditions of the procedure day after day.

For this reason, the present summary is intended not only to record professional observations but also to represent a way of thinking: that even established routine practice must be reconsidered when it does not serve the patient's greatest safety. In this sense, this work also carries the message of Ignác Semmelweis: not simply that hands must be washed, but that we must be willing to go against entrenched habits when they place the patient at risk.

## CRediT authorship contribution statement

**Erzsébet Horváthné Konya:** Writing – review & editing, Writing – original draft, Supervision, Methodology, Investigation, Conceptualization. **Zoltán Balogh:** Writing – review & editing.

## Ethical approval

All observations presented in this work originated from routine nursing practice and did not involve patient enrolment, collection of patient data, or experimental manipulation. The observations were derived from the everyday practice of professional nurses within their normal scope of clinical duties. In accordance with national and institutional regulations, ethical approval was therefore not required. The experiences described are reflective accounts based on professional clinical activities and are shared to support safer and more standardised nursing practice in urinary catheterisation. The reflections and examples were anonymised, and no identifiable clinical or personal details were retained. The authors affirm that the material presented is entirely original, has not been published elsewhere, and was developed solely for the purpose of contributing to professional dialogue in infection prevention and nursing quality improvement.

## Declaration of AI and AI-assisted technologies in the writing process

During the preparation of this manuscript, the author used ChatGPT (OpenAI) to assist with linguistic clarity, structure, and scientific phrasing. The tool was employed solely to enhance language precision and coherence; all conceptual content, observations, and professional interpretations originate from the author's independent work. Following the use of the AI tool, the author reviewed, verified, and edited all text, taking full responsibility for the final version and its accuracy.

## Funding sources

This publication received no specific grant from any funding agency in the public, commercial, or not-for-profit sectors. The work was carried out as part of the authors' professional and academic roles within Semmelweis University, Faculty of Health Sciences.

## Conflict of interest statement

The authors declare that there are no financial or personal conflicts of interest that could have influenced the work reported in this article. No external funding, industry support, or sponsorship was received for the preparation of this paper. The devices and practices discussed in the text are described conceptually and not as commercial products.
